# Lower Extremity Cutaneous Lesions as the Initial Presentation of Metastatic Adenocarcinoma of the Colon

**DOI:** 10.1155/2012/989104

**Published:** 2012-03-26

**Authors:** Dhyan Rajan, Mitanshu Shah, Pooja Raghavan, Shanza Mujeeb, Sadat Rashid, Aieska Desouza, Paul Mustacchia

**Affiliations:** ^1^Department of Internal Medicine, Nassau University Medical Center, East Meadow, NY 11554, USA; ^2^Department of Internal Medicine, Mount Carmel Medical Center, Columbus, OH 43222, USA; ^3^Department of Gastroenterology, Nassau University Medical Center, East Meadow, NY 11554, USA

## Abstract

Cutaneous metastases from colorectal cancers are rare and are usually present on the abdominal wall or previous surgical incision sites. Remote cutaneous lesions have been reported, however, often occur in the setting of widespread metastatic disease including other visceral secondaries. We present a case of lower extremity cutaneous metastases as the first sign of metastatic disease in a patient with adenocarcinoma of the colon. This case illustrates that new skin lesions may be the initial presentation of metastatic disease in a patient with a history of cancer.

## 1. Introduction

Cutaneous metastases from colorectal cancers are rare, occurring in less than 4% of patients [1, 2]. The most common sites of cutaneous spread include the abdominal wall and surgical incision sites [1–3]. Although remote lesions including those on the lower extremities, hands and face have been reported; these lesions often occur in the presence of widely spread metastatic disease [3]. We report a case of a 36-year-old male with metastatic nodules on the lower extremities from an adenocarcinoma of the colon as an initial presentation of extranodal tumor spread.

## 2. Case Presentation

A 36-year-old Hispanic male with no significant past medical history presented to the emergency department with complaints of intermittent hematochezia and an unintentional weight loss for nearly 2 months. He stated that he began noticing bright red blood intermixed with well-formed stool; also admitted to a 26 pound unintentional weight loss over 2 months. He denied any constipation, diarrhea, fever, or tenesmus. He denied the use of any medications, illicit drugs, alcohol, and tobacco. Family history was noncontributory, including the absence of any malignancy or gastrointestinal disorders. A colonoscopy performed during admission revealed a 3 × 3 centimeter friable mass in the ascending colon, with biopsies confirming the presence of a moderately differentiated adenocarcinoma. After a right hemicolectomy was performed, no evidence of serosal invasion, lymph node involvement, or distant metastasis was noted; thus the tumor was staged as T2N0M0. The patient was following with his oncologist for this sporadic colorectal adenocarcinoma and was being appropriately screened for cancer recurrence after surgical intervention.

More than 2 years after hemicolectomy, the patient presented to the ambulatory clinic with complaints of a 2-week history of papules and nodules on his lower extremities. He stated that he initially noticed a 2-millimeter nonpruritic papule 3 centimeters above his right knee, which increased to approximately 5 millimeters within the next 24 hours. 48 hours after the appearance of this papule, the patient noticed a rapid onset of numerous follicular papules ranging from 1 to 5 millimeters in size. The lesions were now present on both the left and right lower extremities. He stated the lesions were erythematous, nonpruritic, nontender, and immobile. He denied the presence of any other lesions, unintentional weight loss, fever, or chills.

**Figure 1:**
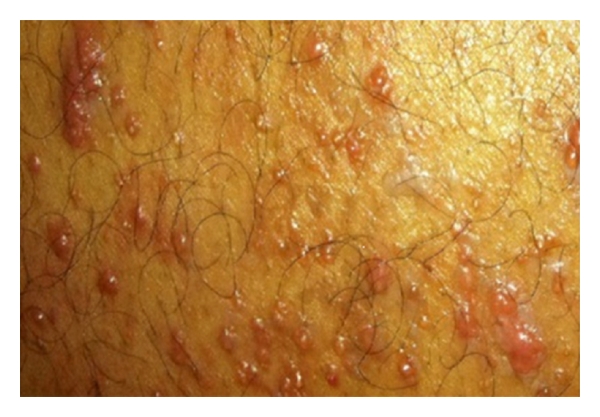
Multiple, erythematous, lesions of varying size present on the skin of the left lower extremity.

**Figure 2:**
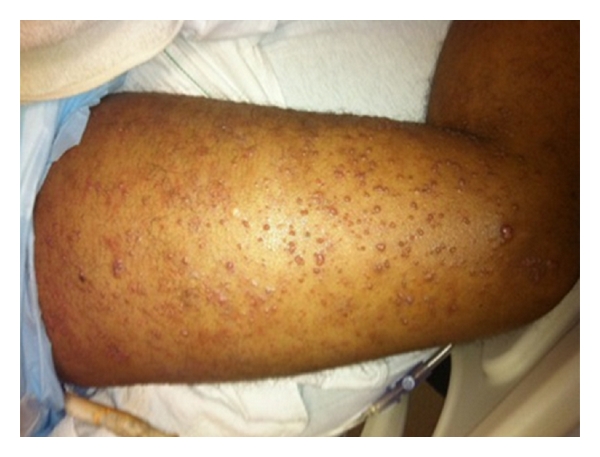
Erythematous follicular papules sometimes coalescing and forming nodules on the patient's right lower extremity.

Physical examination was remarkable for multiple, shiny, immobile, erythematous, follicular papules ranging from 1 to 5 millimeters in size on his bilateral lower extremities (Figures 1 and 2). Some of the lesions also appeared to be coalescing and forming nodules, with the largest nodule being 9 millimeters in size located on the left lower extremity. No lesions were present on the rest of the patient's body including the abdominal wall and the hemicolectomy surgical incision site.

**Figure 3:**
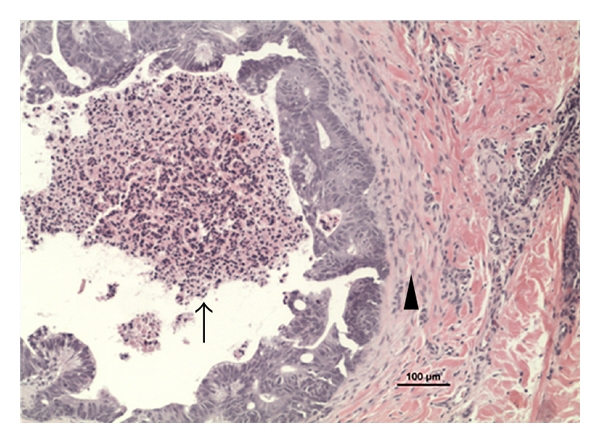
Hematoxylin and eosin staining of a 4 millimeter papule on the right lower extremity. Note the glandular structure of metastatic adenocarcinoma (arrow) with a desmoplastic reaction in surrounding tissue of the dermis (arrowhead).

A 3-millimeter punch biopsy of a 4-millimeter papule on the right lower extremity was performed. The location of the papule was approximately 6 inches above the right knee. Histopathologic evaluation of the biopsy revealed the presence of glandular structures suggesting metastatic adenocarcinoma consistent with a primary colon cancer, with a desmoplastic reaction in surrounding tissue of the dermis (Figure 3). Subsequent bone scintigraphy and computed tomography (CT) of the head, thorax, abdomen, and pelvis revealed no metastatic lesions. After the diagnosis of metastatic disease was made, the patient refused any adjuvant therapy or further diagnostic studies including repeat biopsies.

3 months after initial biopsy, the patient returned to the medical emergency department with shortness of breath and pleurisy. Physical examination was significant for unresolved papular lesions on the lower extremities. The lesions were unchanged in size and number when compared to physical examination 3 months prior. Further examination was also significant for decreased breath sounds in the bilateral lungs. Imaging studies performed at that time revealed multiple lung masses with a histological confirmation of metastatic adenocarcinoma. The patient was intubated for respiratory failure and admitted to the critical care service. His hospitalization was complicated by massive hemoptysis, sepsis, and multiple organ failure leading to his eventual death on hospital day 12.

## 3. Discussion

The occurrence of cutaneous metastasis from internal malignancies is rare, being discovered in only 0.2–10% of autopsies performed on patients with cancer [1, 2]. The frequency of cutaneous metastasis from an internal malignancy is dependant on the type of primary tumor present. In a study by Lookingbill et al., the most common nondermatological tumors that metastasize to the skin are breast and lung cancers; with cutaneous metastases from a breast primary occurring in up to 24% of all patients during the course of their disease [1, 3, 4]. Cutaneous metastases from colorectal cancers are infrequent, accounting for less than 5% of all cutaneous secondaries [5].

Colorectal cancer metastasizing to the skin is rare, with the more common sites of disseminated disease being the liver, lung, bone, and brain [6]. In a study done by Tan et al., more than 2500 patients with colorectal cancer were followed, in which only 3 (0.1 percent) developed cutaneous metastases [7]. Several mechanisms by which cutaneous metastasis from colorectal primaries occur have been postulated. One important mechanism thought to be involved is dermal invasion through intravascular and intralymphatic spread [5, 6]. Direct extension via surgical tracts appears to be another important factor, as reports of cutaneous metastasis overlying the abdominal wall, colostomy sites, and surgical incisions have been reported [5, 8]. In a cohort study of 413 patients with metastatic colorectal cancer, less than 4.5 percent had cutaneous metastasis, with the vast majority of these lesions appearing on the abdominal wall, mostly in abdominal incision sites [4, 9, 10].

Although previous surgical scars and skin overlying the abdominal wall appear to be the most frequent sites of cutaneous metastasis from colorectal cancer, remote lesions have also been reported. Lesions involving the face, tongue, head, neck, and hands have been documented; however, these are usually associated with other visceral secondaries and are likely due to hematogenous spread of tumor cells and their trapping to capillary beds of the overlying skin [9]. Remote lesions as an initial presentation of metastatic disease are rare [10].

The clinical presentation of cutaneous metastasis is varied, posing a diagnostic challenge to clinicians. Typically, lesions present as multiple, firm, nonulcerating nodules which may mimic lipomas, neurofibromas, and granulomas [10]. Lesions have, however, been described as solitary with varying morphological patterns [1, 11]. Varying morphological patterns that have been reported include carcinoma erysipelatoides, alopecia neoplastica, cicatrical, and zosteriform metastasis [2]. Clinicians should also be aware that cutaneous metastatic lesions may invoke a rapid surrounding inflammatory process, mimicking the appearance of cellulitis or folliculitis [12]. Patients with this presentation and a history of cancer should be closely followed up, as this apparent benign dermatologic condition may be camouflaging a more morbid condition such as metastatic disease [7, 12].

Although cutaneous metastasis from colorectal cancer alone poses no medical harm, they often herald widespread metastatic disease and harbor a poor prognosis [2]. Survival after diagnosis of cutaneous metastasis from colorectal cancers is short, and an average survival is 18 months [4, 10].

## 4. Conclusion

Cutaneous metastasis from colorectal cancer is rare and may be the initial sign of metastatic spread. The case described illustrates that clinicians should maintain a heightened suspicion for metastatic disease in a patient with a history of cancer and new, evolving, or unresolving skin lesions [11–13]. Early recognition of such lesions may help in accurate staging of the disease and enable appropriate medical management before widespread metastases occur [10].
